# On the Employment of the Horse-Chestnut in Pulmonary Emphysema in the Horse1Report of the chairman of a commission appointed to inquire into the therapeutic value of the horse-chestnut in pulmonary emphysema, before the Veterinary Society in Paris, at its meeting, May 9, 1895.

**Published:** 1895-10

**Authors:** M. H. Benjamin


### ON THE EMPLOYMENT OF THE HORSE-CHESTNUT IN PULMONARY EMPHYSEMA IN THE HORSE.[1]

BY M. H. BENJAMIN.

A Polish physician residing at Azayle-Freon (India) bought a mare in the month of September, 1892. Some time later M. Cantiget, called in consultation, diagnosed pulmonary emphysema, and advised the use of the horse-chestnut. Six months later a very manifest modification of the movements of the flank was noticed, and the owner said that the cough was very much less frequent. The mare has since continued in an excellent state of health, has a very good respiration for an animal fifteen years old, and only rarely coughs. M. Cantiget afterward made five observations, which are subjoined.

Observation I. M. D., an old bailiff, residing in Prenilly, owned a mare, eighteen years old, which had been in his service thirteen years. At the age of five this mare was attacked with a very grave angina. For some time she had each year an acute attack of the larynx and perhaps of the bronchi.

At the age of ten years, after great exertion in excessive heat, she had an attack of pulmonary congestion. Since this time the symptoms of pulmonary emphysema have been manifest; the cough, above all, was very frequent, and all of the remedies known only served to palliate for a relatively short time. Finally it came to such a point that in December, 1893, it was decided to kill the mare, as she was able to trot only five hundred metres without resting. When consulted I advised the use of the horse-chestnut.

At the beginning of the treatment the emaciation was very

[1] Report of the chairman of a commission appointed to inquire into the therapeutic value of the horse-chestnut in pulmonary emphysema, before the Veterinary Society in Paris, at its meeting, May 9, 1895.

pronounced, the respiratory movements accelerated, and accompanied by a very strong jerking movement, shaking the whole body both in the inspiratory and expiratory movements.

By auscultation one establishes a diminution of the respiratory murmur and the presence of dry and sibilant râles. Percussion denotes an increased air sound.

If made to trot but a few metres the respiration becomes precipitous and the nostrils greatly dilated. In the stable the respirations were 24 and the pulsations 42.

Notwithstanding the gravity of these symptoms the appetite remained good.

On the 20th of October it was determined to give the animal from 100 to 300 grammes daily of horse-chestnuts cut fine and mixed with some bran, without further change of her daily food. The medication was commenced by giving 100 grammes and gradually increasing the dose.

A month later the respirations were only 16 and the pulsations 33 to the minute, and in the month of March, 1894, respirations 11 and pulsations 35. The general condition improved, the cough became very slight; the respiratory movements were irregular, it is true, but the jerking of the flank much less pronounced, above all, during inspiration. Auscultation established, the disappearance of the râles and the respiratory murmur greatly diminished. It was possible to drive this mare twelve kilometres in an hour without fatigue, and at the end of this exercise the flank movement had been augmented but slightly.

At the end of March this medication was stopped, but no other treatment was employed. Occasional embarrassments in the respiration was noted, which lasted for some hours, but this short-lived discomfort disappeared when an expectorating bronchitis was produced.

In the month of October, 1894, the horse-chestnut medicament was again resorted to. At this time the respirations were 18 and the pulsations 38. On the 14th of last December I noted respirations 14, pulsations 36. The animal is always in good health, coughs rarely, and does not fatigue *en route*.

Observation II. M. B. is neighbor to the proprietor who owns the mare previously described. This animal has also been the subject of attack for several years. She is now about twelve years old.

The employment of arsenious acid, digitalis, belladonna, and turpentine gave only the most transient results.

In the month of January she was submitted to daily doses of 250 grammes of finely cut horse-chestnuts. At this time the respirations were 20 and the pulsations 38 at repose in the stable.

Two months later the animal had a severe attack of indigestion, and the owner attributed the cause of the attack to the ingestion of horse-chestnuts, while already some important results had been obtained. In effect, the flank movement became more regular, respirations 13, and pulsations 36 to the minute. The crepitant râles could no longer be heard, and the cough was scarcely perceptible.

In October, 1894, the treatment was resumed. At this time the respirations were 18 and the pulsations 36. On the 18th of December the respirations were 12 and the pulsations 36.

Observation III. A small horse, about six years old, slowly attacked with pulmonary emphysema. Usual symptoms—respirations 26, pulse 60. Treatment the same as above. Three months later symptoms very much diminished. Respirations 20, pulsations 50. The irregularity in the movement of the flank always remained, but was less pronounced. The animal coughed only rarely.

The treatment was suddenly interrupted on account of circumstances. I was resumed in October, 1894. At this time the respirations were 18 and the pulsations 50. On the 18th of December the respirations were 16 and the pulsations 55. The amelioration established at first was continued.

Observation IV. In October, 1894, a strong mare, sixteen years of age, unable to perform rapid service, was placed under treatment. Respirations after exertion, 40 per minute; at rest, 22. Pulsations 45. Flank movements irregular, both on inspiration and expiration. Aggravated dry cough, both at work and in the stable. Auscultation established a diminution of the respiratory murmur and the presence of dry, crepitant râles.

On the 16th of December last the number of respirations was 18 and pulsations 40. The cough and irregular movements of the flank remain, but these symptoms are diminished in intensity. Perhaps the dose of 100 grammes given daily to this mare, which is of large size, was insufficient.

Observation V. A mare which had the peculiar cough of broken wind in the stable, but which did not cough in driving, and which presented no other symptoms of heaves, under this treatment coughed very much more rarely.

M. Cantiget concludes his memoirs with some considerations of the horse-chestnut tree, and on the chemical composition of its fruit. It remains to be determined what the principle is which, from a therapeutic point of view, is so efficacious in ameliorating the symptoms of pulmonary emphysema in the horse.

M. Cagney and myself have seen the proprietors of the horses mentioned in Observations I., II., III., IV. They are enthusiastic over the results. Their animals are certainly much improved. Their respiration is scarcely more accelerated at the trot than in repose. They all work daily, and M. D. tells us that his mare has trotted a dozen kilometres in forty-five minutes. He adds that he has continued to give the mare the finely cut horse-chestnut for the past two years.

M. B., recorded in Observation II., showed us how damp his stable was. Nevertheless, the animal is in good health, and eats hay every day. His respirations are 20.

The pony, the subject of Observation III., has always had the characteristic cough, but is very vigorous and in good health.

The mare in Observation IV. has 26 respirations per minute. She is also in good health, and serves amply for the quick work required in a butcher's wagon.

M. Cantiget concluded with a case of a bay gelding of large size, thirteen years old, which had become, during the last year, emphysematous to the point of being useless. Since being treated he is able to pull heavy loads. There are times when his master ceases for two days to give him his medicine. Then the symptoms of emphysema are accentuated to the extreme until the treatment is resumed. The respirations in repose are 18 per minute.

### Discussion.

M. Saussy.—The study of the active principles of the horse-chestnut have not yet been taken up. The important point to establish is that relative to the possibility of work in the subjects which have been good for nothing.

An interesting article has been published in the *Journal of Veterinary Medicine and Zootechnics*, by M. Cornevin. The learned professor of the Lyons School has experimented with the horse-chestnut on sheep, pigs, and dogs, the duck, with which it is eminently toxic, but his researches have not been carried to the horse. This animal appears to be not incommoded by its use. It may not perhaps be the same with the human species, if I am to judge by the statements of the owner of one of the horses treated. His wife, being very asthmatic, and seeing the good effects produced on his mare, concluded to eat some of the horse-chestnut. She cut it up and mixed it intimately with some powdered sugar. Unfortunately it produced a painful attack of indigestion, with nausea, vomiting, and general malaise, which took away all desire to try it again.—*Receuil de Médecine Vétérinaire*, May 30, 1895.

